# Commentary: Toward a new focus in antibiotic and drug discovery from the *Streptomyces arsenal*

**DOI:** 10.3389/fmicb.2015.00727

**Published:** 2015-07-16

**Authors:** Dipesh Dhakal, Jae Kyung Sohng

**Affiliations:** Institute of Biomolecule Reconstruction (iBR), Department of BT-Convergent Pharmaceutical Engineering, Sun Moon UniversityAsan, South Korea

**Keywords:** antibiotic resistance, *Streptomyces*, synthetic biology, combinatorial biosynthesis, mutasynthesis

Infectious diseases caused by bacteria, particularly those gaining drug-resistance, are among the top causes of mortality in the world (Overbye and Barrett, 2005). The mechanisms of multidrug efflux systems, enzymatic modification and inactivation of drug molecules have enabled the resistant bacteria to reduce the potency of common antibiotics (van Hoek et al., 2011; Lin et al., 2015). Thus, the discovery of new antimicrobials and expansion of utility of existing antibiotics by overproduction or targeted modification is crucial to combat the ever-increasing antimicrobial resistance (Dhakal et al., 2015; Lin et al., 2015).

*Streptomyces* is the major sources of natural products including effective antimicrobials (Chaudhary et al., 2013). Antoraz et al. (2015) have summarized advances on drug discovery from *Streptomyces arsenal*, illustrating different approaches for elicitation of antimicrobials by nutritional and hormonal signals or production of useful antimicrobials by co-culture and *in situ* culture technologies. The authors have flash-lighted on utility of different synthetic biological and system biological based metabolic engineering techniques for harnessing the biosynthetic capabilities of *Streptomyces*. It can give rise to superfluous possibilities for antibiotic discovery by using these precise genetic engineering techniques.

Genome mining has revealed that *Streptomyces* can harbor numerous active or cryptic biosynthetic gene clusters encoding for diverse compounds including novel antimicrobials (Chaudhary et al., 2013). Fundamentally, identification of novel antimicrobials can be achieved by high throughput screening techniques, either (a) compound specific screening or (b) organism specific screening (Figure 1A). In the first approach, the structurally characterized molecule is tested against different pathogenic organisms. In next approach, the pathogenic organism is screened against different putative compounds and effects are assessed. Furthermore, bioinformatics tools assisting on studies rendering to structure-activity relationships (SAR) or quantitative structure activity relationship (QSAR) can assist in developing the effective drug molecules from microbial resources.

**Figure 1:**
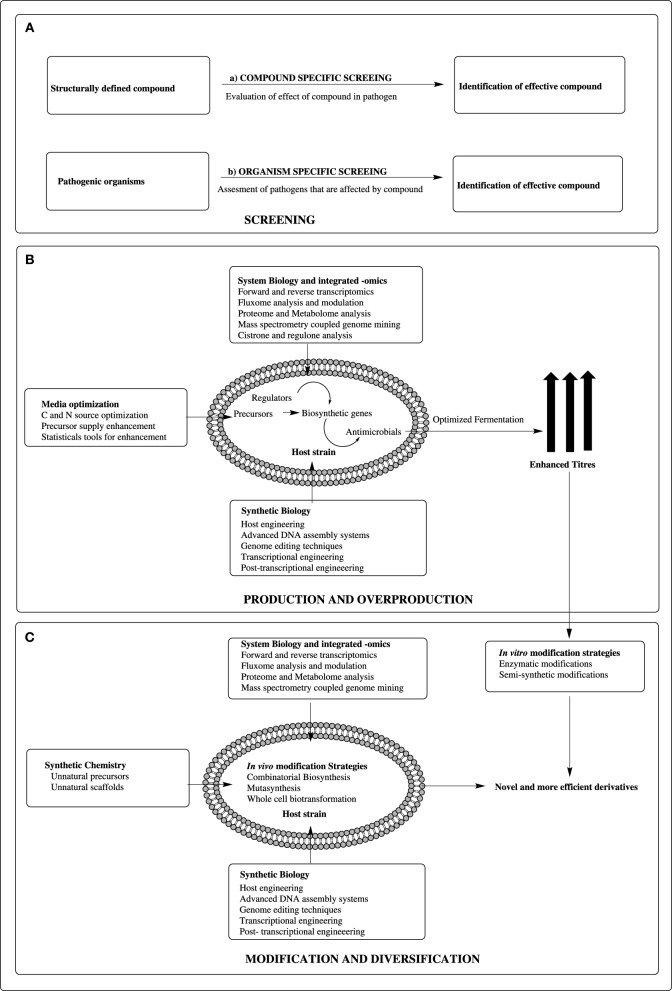
**Integrated approach for exploiting** ***Streptomyces arsenal*** **to combat the antibiotic resistance. (A)** Extensive screening strategies for identifying lead antimicrobials: (a) Compound specific screening (b) Pathogenic organism specific screening. **(B)** Production and overproduction strategies. The knowledge of synthetic biology, integrated-omics (genomics, proteomics, metabolomics, etc…) and system biology can be used for developing efficient production host. The performance of host can be further ameliorated with media optimization and fermentation technology for optimum production. **(C)** Compound modification and diversification strategies. By using knowledge of synthetic chemistry, synthetic biology, system biology and integrated-omics, various novel and effective derivatives can be developed using *in vivo* and *in vitro* approaches.

Antoraz et al. (2015) indicated that the application of bioinformatics and “-omic” based engineering have important contribution in drug discovery. The key focus of these technologies is production and overproduction of important molecules or their structural/functional diversification for pharmaceutical value. The precise knowledge of biochemistry of secondary metabolites production supported with advanced system biological and integrated “-omic” techniques provide rational strategies for production and overproduction of targeted compounds in native/heterologous hosts (Gomez-Escribano and Bibb, 2011; Chaudhary et al., 2013; Hwang et al., 2014). The production profile can be modulated by interrogation with media optimization or medium component selection strategies (Jose et al., 2013). The application of synthetic biological tools such as construction of a complete genetic circuit or rewiring the transcriptional or post-transcriptional regulation, play significant role in production and overproduction strategies (Medema et al., 2011; Wohlleben et al., 2012; Weber et al., 2015) (Figure 1B). The optimization of bioprocessing and fermentation technology may help in harnessing the maximum yield of the desired compound. In addition, for compounds that are cryptic and not amenable to production from native host, a stable chassis can be utilized as heterologous host. The host can in turn be rationally engineered for maximum production using synthetic biological platform.

Antoraz et al. (2015) illustrated that diverse synthetic biological approaches can be efficient method for precise modifications to attain a new molecules with better antimicrobial activities. These synthetic biological techniques in synergy with system biological impetus can be employed in two categories (a) living cell based (*in vivo*) or (b) chemical reagents based (*in vitro*) techniques (Figure 1C). The generation of natural product-derived libraries is assisted by synthetic chemistry, where novel structural diversity is generated by precursor directed biosynthesis or mutasynthesis or semi-synthesis approaches (Kirschning et al., 2007; Kennedy, 2008). In precursor-directed biosynthesis, the synthetic analogs of some scaffold moieties are fed and incorporated during metabolite production in wild type strains generating novel analogs. Mutasynthesis is a refined version of precursor directed biogenesis, where the producer strain is engineered curtailing the competitive pathways, yielding maximum production of desired compounds. In semi-synthesis, the designed chemical moiety is attached to the natural product by specific chemical reactions. In another approach termed “combinatorial biosynthesis,” the substrate promiscuity of natural or engineered enzymes and modulated pathways is utilized to produce “unnatural products” with potential pharmaceutical value (Sun et al., 2015). This approach can be employed by altering the precursors or enzyme level modifications through mutations. The domain alterations or complete pathway level recombination is another approach for combinatorial biosynthesis. In addition, the whole cell based *in vivo* biotransformation or reaction tube based *in vitro* catalysis contribute for generation of a repertoire of novel derivatives of existing antimicrobials (Figure 1C).

It seems we are running out our defensive options due to alarming increase in the drug resistant bug in contrast to decrease in the introduction of new antimicrobials. There is skepticism regarding potential post-antibiotic era when common infections may lead to mortality because of dwindling antibiotic arsenal. Antoraz et al. (2015) have illustrated the profound potential of *S. arsenal* as prolific source of antibiotics and drug discovery. Moreover, there are sufficiently availed details of biochemistry and physiology behind the pathogenicity of resistant bacteria as well as the biosynthetic ability of antimicrobial producers. Hence, it can be hoped that more efficacious antimicrobials can be developed from *Streptomyces* on the bases of all the available knowledge resources and technologies.

## Conflict of interest statement

The authors declare that the research was conducted in the absence of any commercial or financial relationships that could be construed as a potential conflict of interest.
